# Association of prehospital invasive blood pressure measurement and treatment times of intubated patients with suspected stroke – a retrospective study

**DOI:** 10.1186/s13049-025-01411-5

**Published:** 2025-05-16

**Authors:** Michael Eichlseder, Nikolaus Schreiber, Alexander Pichler, Michael Eichinger, Sebastian Labenbacher, Barbara Hallmann, Simon Orlob, Paul Zajic, Simon Fandler-Höfler

**Affiliations:** 1 Division of Anaesthesiology and Intensive Care Medicine 1, Medical University of Graz, Auenbruggerplatz 5, 8036 Graz, Austria; 2 Division of Anaesthesiology and Intensive Care Medicine 2, Medical University of Graz, Graz, Austria; 3 Institute of Emergency Medicine, University Hospital Schleswig-Holstein, Kiel, Germany; 4 Department of Neurology, Medical University of Graz, Graz, Austria

**Keywords:** Emergency medical services, Blood pressure, Arterial pressure, Stroke, Hemodynamic monitoring, Anesthesia, Intubation

## Abstract

**Background:**

Invasive blood pressure measurement is commonly used in in-hospital patients with stroke requiring general anesthesia, but is much less established in the prehospital setting. While it allows for more precise blood pressure management, it might also lead to prehospital treatment delays. Therefore, this study aims to evaluate the potential impact of prehospital invasive blood pressure measurement on treatment times.

**Methods:**

Adult patients (≥ 18 years) with suspected stroke (both ischemic and hemorrhagic) and prehospital induction of emergency anesthesia by physicians admitted to the University Hospital of Graz between January 1st, 2018 and December 31st, 2023, were included. Optimal one-to-one matching using a propensity score for prehospital invasive blood pressure measurement based on patient age, patient sex, treatment by helicopter emergency medical services and Glasgow coma scale on scene was performed. Primary outcome was the time-interval between on-scene arrival of the prehospital physician and first cranial computed tomography (CCT).

**Results:**

One hundred patients with suspected stroke and prehospital emergency anesthesia were identified, of whom 67 (67%) had prehospital invasive blood pressure measurement. After matching, 33 patients of each cohort were used for main analysis. Median (25th to 75th percentile) time between on-scene arrival and first CCT was 79 (70–87) minutes in the prehospital measurement group, compared to 73 (67–81) minutes in the group with in-hospital initiation of invasive measurement (*p* = 0.21). On-scene time was longer in the prehospital group [45 (37–51) vs. 36 (33–43) minutes, *p* = 0.009], while transport duration [18 (11–25) vs. 20 (13–31) minutes, *p* = 0.20] and time spent in the resuscitation room [16 (12–20) vs. 16 (12–21) minutes, *p* = 0.391] did not differ.

**Conclusion:**

In summary, among patients with suspected stroke who underwent prehospital intubation, time from on-scene arrival to the first CCT was not prolonged in those who received prehospital invasive blood pressure measurement compared to those who received it in-hospital.

## Background

Stroke constitutes one of the most frequent emergencies, putting a major burden on patients, societies and healthcare systems alike. With more than 12 million people having a stroke annually and 6.5 million deaths attributed to stroke every year, optimal care is of utmost importance [1–3]. Suspected strokes commonly lead to an activation of prehospital emergency systems, with up to one tenth of the missions being due to a neurological origin [4].

Ensuring stable cardiorespiratory conditions and facilitating a prompt transfer to a hospital with the ability to perform disease-specific diagnostics and interventions are the primary objectives during the prehospital phase. Both ischemic and hemorrhagic stroke can lead to a reduction in consciousness. In these patients, maintaining adequate oxygenation and carbon dioxide elimination may necessitate the administration of prehospital emergency anesthesia to secure the airway and facilitate individualized ventilatory support [5, 6]. Induction of emergency anesthesia, however, is associated with hemodynamic disturbances [7, 8]. Special attention is required due to the common occurrence of blood pressure variations in the early phase following a stroke. Both severe hypotension and hypertension, with varying ranges between ischemic and hemorrhagic stroke, must be strictly avoided [9–12].

Consequently, invasive blood pressure monitoring, enabling beat-to-beat analysis and rapid pharmacological intervention, is considered the gold standard in the in-hospital management of unconscious patients with stroke [13]. In the prehospital setting, however, arterial cannulation and invasive blood pressure measurement are only occasionally performed. Arguments against this practice are the required skill level, necessary equipment, and potential delays to diagnostics and treatment. Whether the latter actually holds true is however unknown.

This study aims to investigate if there is a difference in treatment time between patients with prehospital arterial cannulation and invasive blood pressure measurement in comparison to patients having it performed in-hospital.

## Methods

This manuscript was written according to the Strengthening the Reporting of Observational Studies in Epidemiology (STROBE) guideline [14].

### Study design and setting

This study was a single-center, retrospective analysis of routinely collected data. The University Hospital of Graz is a tertiary academic center. As a comprehensive stroke center, care is provided for patients with both ischemic and hemorrhagic stroke, covering approximately 1.500.000 individuals. Patients without prehospital airway management are primarily seen by neurologists in the emergency department. Whenever patients already receive prehospital induction of emergency anesthesia or are defined as critical by the prehospital team, they bypass the regular emergency department and are treated in the resuscitation room by a team of anesthesiologists and neurologists. Prehospital emergency anesthesia is solely performed by prehospital physicians, who staff both ground-based response units and helicopter emergency medical services (HEMS). They undergo specific prehospital training in addition to their background specialty (mostly anesthesiology, internal medicine or intensive care medicine) and are dispatched to severe emergencies potentially requiring advanced medical treatment on-scene together with paramedics. Units are equipped for arterial cannulation and invasive blood pressure measurement, which can be performed at physicians’ discretion [15].

### Selection of participants

Admission logs from the resuscitation room were screened for adult patients (≥ 18 years) admitted due to an acute neurological cause between 1 st January, 2018, and 31 st December, 2023. All patients fulfilling the following criteria were included: primary mission (no transfers from other hospitals), prehospital induction of emergency anesthesia and airway management, and stroke (ischemic and/or hemorrhagic) suspected by the prehospital team.

If patients were treated by two or more prehospital physician systems (e.g., ground-based initial treatment and subsequent transport by HEMS), if no cranial computed tomography (CCT) directly after initial treatment in the resuscitation room was performed, or if data regarding the primary outcome was missing, patients were excluded. Finally analyzed patients were divided into two groups (prehospital invasive blood pressure measurement group and in-hospital invasive blood pressure measurement group), according to the time point of invasive blood pressure measurement initiation.

### Measurements

Initiation point of invasive blood pressure measurement, either prehospital or in-hospital, was determined by reviewing prehospital records and resuscitation room documentation. Demographic and case-specific data (age, sex, suspected diagnosis), time-points (time of on-scene arrival, time of transport start, handover time, time of first CCT), Glasgow coma scale (GCS) at arrival on-scene, vasopressor application, and transport mode were extracted from the prehospital record, resuscitation room record, and hospital record.

### Outcomes

The primary outcome was the time-interval between on-scene arrival of the prehospital physician and first CCT. Secondary endpoints were time between on-scene arrival of the prehospital physician and handover in the resuscitation room, time on-scene of the prehospital physician, duration of transport, and time between handover in the resuscitation room and first CCT. Additionally, exploratory outcomes were systolic and diastolic blood pressure resuscitation room arrival.

### Matching

To mitigate implications of physician’ decision making (e.g., baseline patient characteristics, degree of impairment of consciousness) on group balancing and emulate a randomized experiment, a propensity score *e* for prehospital invasive blood pressure measurement was calculated using logistic regression. Variables factoring into this propensity score *e* were: age, sex, treatment by HEMS and GCS at arrival on-scene. Using this propensity score, optimal matching using a one-to-one ratio was performed. Covariate balance after matching was assessed using Standardized Mean Differences (SMDs), with SMDs below 0.2 considered not indicative of a potentially relevant magnitude of difference. Missing data were imputed using Multiple Imputation by Chained Equations (MICE) to retain all patients in the propensity score matching.

### Statistical analysis

Demographic, injury-related, and treatment-related data were presented as median and 25 th to 75 th percentile, or number (*n*) and percentages (%), as appropriate. To assess for between-group differences before matching, Wilcoxon rank-sum test (for interval-scale data) or Pearson’s Chi-squared test (for nominal-scale data) were used. To assess for between-group differences after matching, Wilcoxon signed-rank test was used. *P*-values below 0.05 were considered significant.

Matching and all statistical analyses were conducted using R version 4.4.0 (The R Foundation for statistical computing, Institute for Statistics and Mathematics, Vienna University of Economics and Business, Vienna, Austria), with the MatchIt and MICE packages, alongside base R functions.

## Results

**Fig. 1:**
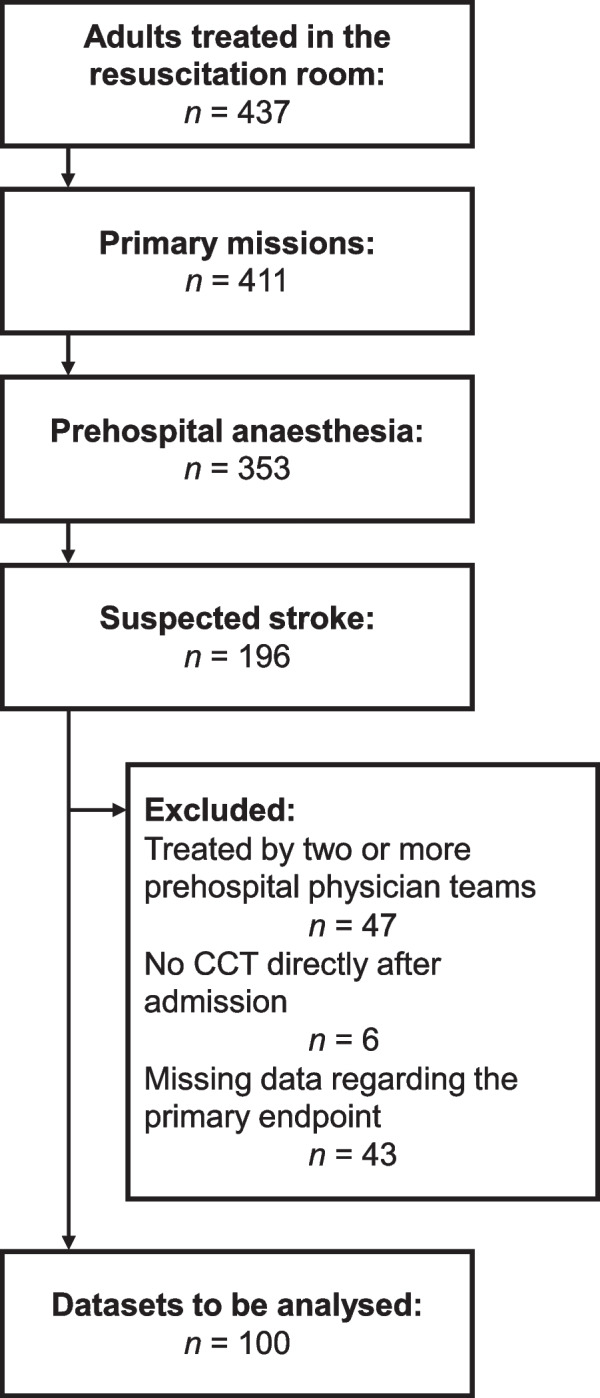
Study flow chart. CCT = cranial computed tomography

In the selected timespan, 437 adult patients were treated in the resuscitation room for an assumed acute neurological cause. Of those, 196 were primary missions with a suspected stroke who received prehospital induction of emergency anesthesia and airway management. After exclusion of 47 patients who were treated by two or more prehospital physician systems, six without an immediate CCT, and 43 patients with missing data regarding the primary endpoint, 100 patient data sets were available for analyses (Fig. 1). Of those, 67 received prehospital invasive blood pressure measurement, with the remaining 33 having invasive blood pressure measurement initiated in the hospital.

Overall, median (25 th to 75 th percentile) patient age was 72 (61–80) years, 57% were male, and patients were mostly transported by ground-based ambulance (75/100, 75%). Further baseline patient characteristics are presented in Table 1. Datapoints were missing in four patients (twice age and twice GCS at arrival on-scene) and imputed as described in the methods section.

**Table 1:** Study population characteristics

Characteristic	Overall	In-hospital IBP, unmatched sample	Prehospital IBP, unmatched sample	In-hospital IBP, matched sample	Prehospital IBP, matched sample
n	100	33	67	33	33
Age [years] – median (25 th to 75 th percentile)	72 (61–80)	71 (62–79)	72 (59–81)	71 (62–79)	72 (61–80)
Male sex – n (%)	57 (57)	21 (64)	36 (54)	21 (64)	19 (58)
Initial Glasgow Coma Scale – median (25 th to 75 th percentile)	5 (4–7)	5 (4–7)	5 (4–7)	5 (4–7)	5 (4–7)
Mode of Transport – n (%)					
Ground based	75 (75)	21 (64)	54 (81)	22 (67)	24 (73)
HEMS	25 (25)	12 (36)	13 (19)	11 (33)	9 (27)
Prehospital vasopressor usage – n (%)	49 (49)	17 (52)	32 (48)	17 (52)	16 (48)
Prehospital fluid volume [ml] –median (25 th to 75 th percentile)	500 (500–1000)	500 (500–1000)	500 (500–1000)	500 (500–1000)	500 (500–1000)
In-hospital diagnosis – n (%)					
Hemorrhagic stroke	55 (55)	16 (48)	39 (58)	16 (48)	16 (48%)
Ischemic stroke	19 (19)	7 (21)	12 (18)	7 (21)	7 (21%)
Other	26 (26)	10 (33)	16 (24)	10 (33)	10 (30%)

*HEMS* Helicopter emergency medical service, *IBP* invasive blood pressure, *ml* millilitre

One-to-one propensity-score matching yielded two groups of 33 patients each and acceptable balance measures (Table 2).

**Table 2:** Standardized Mean Differences of the variables before and after matching

Characteristic	In-hospital IBP, unmatched sample	Prehospital IBP, unmatched sample	SMD (unmatched)	In-hospital IBP, matched sample	Prehospital IBP, matched sample	SMD (matched)
n	33	67		33	33	
Age [years] – median (25 th to 75 th percentile)	71 (62–79)	72 (59–81)	0.0187	71 (62–79)	72 (61–80)	0.0340
Male sex – n (%)	21 (64)	36 (54)	0.0991	21 (64)	19 (58)	0.0606
Initial Glasgow Coma Scale – median (25 th to 75 th percentile)	5 (4–7)	5 (4–7)	−0.3064	5 (4–7)	5 (4–7)	−0.1519
Mode of Transport – n (%)			−0.1696			−0.0303
Ground based	21 (64)	54 (81)		22 (67)	24 (73)	
HEMS	12 (36)	13 (19)		11 (33)	9 (27)	

*HEMS* helicopter emergency medical service, *IBP* invasive blood pressure, *SMD* Standardized Mean Difference

**Fig. 2:**
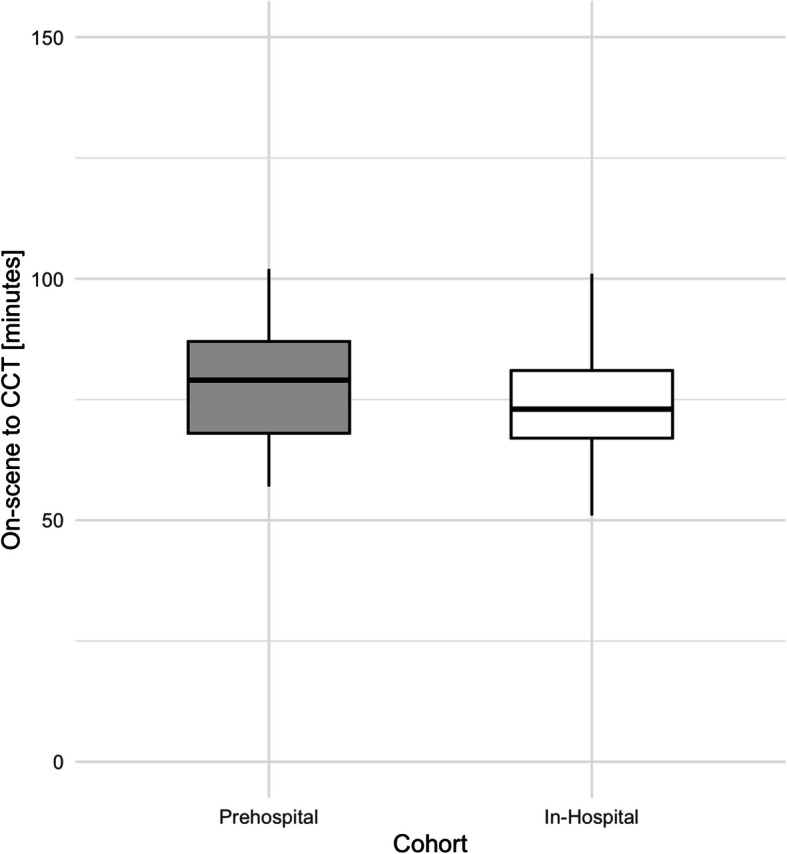
Boxplot comparison of the time-interval between on-scene arrival and the first CCT after hospital admission between the prehospital and in-hospital invasive blood pressure measurement groups. CCT = cranial computed tomography

**Fig. 3:**
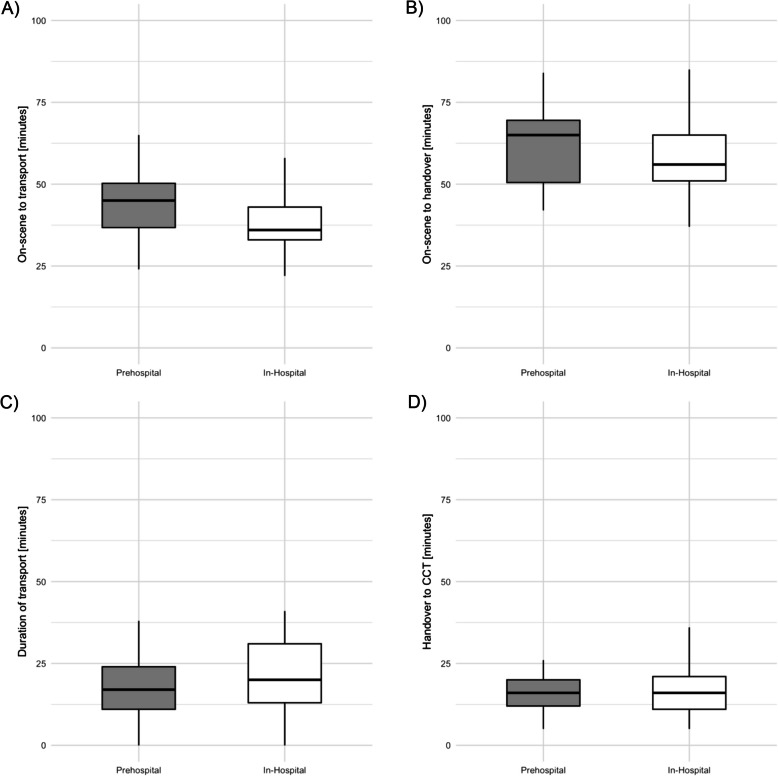
Boxplot comparisons of the time-interval between (**A**) on-scene arrival and transport, **B** on-scene arrival and hospital handover, **C** transport and hospital handover, and (**D**) hospital handover and first CCT after hospital admission between the prehospital and in-hospital invasive blood pressure measurement groups. CCT = cranial computed tomography

In the matched cohort, median time between on-scene arrival and first CCT was 79 (77–87) minutes in the prehospital group compared to 73 (67–81) minutes the in-hospital group (*p* = 0.21) (Table 3, Fig. 2). Time spent on scene [45 (37–51) vs. 36 (33–43) minutes, *p* = 0.009] was significantly prolonged in the prehospital invasive blood pressure group, while time from arrival on-scene until handover [65 (52–73) vs. 56 (51–65) minutes, *p* = 0.08], duration of transport [18 (11–25) vs. 20 (13–31) minutes, *p* = 0.20] and time spent in the resuscitation room [16 (12–20) vs. 16 (11–21) minutes, *p* = 0.39] were not different (Table 3, Fig. 3).

**Table 3:** Median duration in minutes of the selected time-intervals in the overall, prehospital IBP and in-hospital IBP cohort in the unmatched and matched sample

Characteristic	Overall	In-hospital IBP, unmatched sample	Prehospital IBP, unmatched sample	*p*-value*	In-hospital IBP, matched sample	Prehospital IBP, matched sample	*p*-value**
n	100	33	67		33	33	
On-Scene to CCT [minutes] – median (25 th to 75 th percentile)	79 (67–91)	73 (67–81)	79 (67–93)	0.15	73 (67–81)	79 (70–87)	0.21
On-Scene to handover [minutes] – median (25 th to 75 th percentile)	59 (51–75)	56 (51–65)	65 (50–78)	0.11	56 (51–65)	65 (52–73)	0.08
On-Scene to transport [minutes] – median (25 th to 75 th percentile)	43 (33–51)	36 (33–43)	45 (34–53)	0.01	36 (33–43)	45 (37–51)	0.009
Duration of transport [minutes] – median (25 th to 75 th percentile)	18 (13–26)	20 (13–31)	18 (13–25)	0.53	20 (13–31)	18 (11–25)	0.20
Handover to CCT [minutes] – median (25 th to 75 th percentile)	16 (11–20)	16 (11–21)	17 (12–20)	0.67	16 (11–21)	16 (12–20)	0.39

*CCT* cranial computed tomography, *IBP* invasive blood pressure

^*^Wilcoxon rank-sum test

^**^Wilcoxon signed-rank test

No differences were found upon resuscitation room arrival in systolic [127 (109–150) vs. 130 (110–160) mmHg, *p* = 0.99] and diastolic blood pressure [80 (60–90) vs. 80 (65–90) mmHg, *p* = 0.57].

## Discussion

Time from arrival on-scene until initial CCT in this study was 79 min in patients receiving prehospital invasive blood pressure measurement and 73 min in those who had it initiated in the resuscitation room, revealing no significant difference. However, the on-scene time of the physician response units (45 min vs. 36 min) was prolonged in those with prehospital invasive blood pressure measurement.

Data of on-scene times for prehospital induction of emergency anesthesia in similar systems varies. In a retrospective study from Finland evaluating 4496 patients with both traumatic and non-traumatic indications for prehospital intubation, median on-scene time was 33 (23–45) minutes in case of first pass success and 40 (29–52) minutes if two or more intubation attempts were required [16]. Two Australian studies showed markedly longer on-scene times. In an investigation of patients with stroke (both ischemic and hemorrhagic) and advanced airway management, mean time from arrival on-scene to transport was 58 min [17]. In a more heterogenous group of patients with non-traumatic brain injury, median time on-scene was 77 min [18].

The nominal difference of six minutes in the time from arrival on-scene to CCT is slightly longer compared to previously published data. In a previous study in our system by *Wildner *et al*.*, prehospital arterial cannulation required a median of two minutes and preparation of the invasive blood pressure set, which is usually carried out simultaneously, a median of three minutes [15]. Furthermore, time between arrival on-scene and initial CCT did not differ in patients with isolated traumatic brain injury and on-scene times were prolonged only by a median of three minutes in the prehospital invasive blood pressure measurement cohort [19].

Reasons for this difference remain speculative and the following aspects might play a role in this:

In this study patients suffering from stroke were investigated. Compared to trauma victims, these patients are frequently older and may have pre-existing cardiovascular disease, for example arteriosclerosis. These comorbidities can make arterial cannulation difficult and time-consuming. In addition, a lot of emphasis by emergency medical systems is put on achieving short on-scene times—ideally within one hour—in trauma patients. This focus might be less pronounced in the care of non-traumatic patients.

Interestingly, in the exploratory analysis of the admission blood pressure, no difference between the two groups was found. Improved hemodynamic management due to invasive blood pressure measurement was previously shown in the in-hospital setting: improved hypotension detection and substantial decrease of hypotension during anesthetic induction led to the recommendation to establish invasive monitoring prior to induction in high-risk hospitalized patients [20, 21]. The observed discrepancy may be explained by our study’s methodology, which restricted blood pressure assessment to a single time point during handover in the resuscitation room. At handover, situations with a high risk of hemodynamic disturbances, such as anesthetic induction, have already been performed, and a steady state is commonly achieved. Further, blood pressure measured non-invasively could possibly underestimates the incidence of hypotension [22–24].

The question remains whether prehospital arterial cannulation for invasive blood pressure measurement, despite its association with a prolonged on-scene time in this study, provides sufficient clinical benefit to justify its out-of-hospital use. To fully answer this question, a large prospective randomized trial is necessary. However, the following aspects may potentially support it:

In the study by *Fouche *et al*.*, longer on-scene times were associated with a higher probability of survival in patients with hemorrhagic stroke and prehospital rapid sequence intubation [25]. In contrast, the probability of survival decreased with longer on-scene times in patients with traumatic brain injury. Of course, an association of prolonged on-scene time and improved survival generally seems unplausible and the referenced study has a high risk of bias. However, this could still potentially indicate that there is some room and time for additional advanced prehospital interventions in patients with stroke and airway management providing a benefit despite slight on-scene time prolongation. Invasive blood pressure measurement is the in-hospital gold standard for these patients. This is primarily due to its capacity for rapid interventions facilitated by beat-to-beat analysis and the inherent advantages it offers in measurement accuracy compared to non-invasive methods. The authors of a retrospective study from a HEMS service in the United Kingdom, which concurrently measured invasive and non-invasive blood pressure values, concluded that non-invasive values are frequently inaccurate, particularly in patients with hemodynamic instability, and direct measurement should be considered [22]. A similar conclusion was reported in a study comparing invasive and non-invasive measurements in in-hospital patients with stroke. Patients with systolic blood pressures above 180 mmHg had a mean non-invasive value 19.8 mmHg (95% confidence interval 12.2–27.4) below the invasive measurement [26]. This is, especially in patients having hemorrhagic stroke, of high relevance. In these patients, close blood pressure monitoring with narrow limits is recommended in guidelines [9, 27].

A recent randomized trial evaluated prehospital blood pressure management in patients with stroke and provided neutral outcomes. However, in the subgroup of patients with hemorrhagic stroke, a lower odds ratio for poor functional outcome was shown if strict blood pressure management (aiming at a systolic blood pressure of 140 mmHg) was performed in the prehospital phase compared to usual care (treatment only of systolic blood pressure was above 220 mmHg) [28]. In a retrospective study conducted in Australia, the intracranial blood volume measured in the admission CCT was positively correlated with the prehospital systolic blood pressure in patients with intracranial hemorrhage [29]. At the same time, it has been shown that critical blood pressure drops, which frequently happen during induction of anesthesia, are associated with worse outcomes in patients with ischemic stroke injury [30, 31].

### Limitations

There are several limitations to this study. Due to the retrospective nature and hence absence of randomization, causal inference cannot be drawn and an indication bias might be present. To address this, stringent propensity score matching was performed to mitigate potential biases and strengthen the reliability of the findings. Prehospital systems vary worldwide and therefore, these findings may not be directly translatable to countries with different out-of-hospital structures. Moreover, because only patients with documented arterial access were included, patients with failed cannulation could not be identified, introducing potential bias that could not be accounted for. However, in two studies evaluating arterial access in a prehospital population, unsuccessful cannulation was rare [15, 32]. Hemodynamic parameters obtained in the prehospital setting would be of great interest and offer additional valuable insights. Nevertheless, a direct comparison of prehospital blood pressure measurements was not possible, as not all prehospital records provided the necessary detail and precision required for scientific analysis. Furthermore, the sample size is limited, which has to be accounted for when interpreting the results.

## Conclusion

In summary, in patients with suspected stroke who were intubated prehospitally, time from arrival on-scene to the first CCT was not prolonged in the cohort with prehospital invasive blood pressure measurement compared to the cohort with in-hospital establishment. On-scene time, however, was. Further research evaluating hemodynamic and functional consequences of prehospital blood pressure management is required to put this into perspective.

## Data Availability

The data that support the findings of this study are not openly available due to reasons of sensitivity and are available from the corresponding author upon reasonable request. Data are located in controlled access data storage at the Medical University of Graz.

## Abbreviations

CCT: Cranial computed tomography
GCS: Glasgow coma scale
HEMS: Helicopter emergency medical services
SMD: Standardized Mean Differences
STROBE: Strengthening the Reporting of Observational Studies in Epidemiology
